# α-Glucosidase and Bacterial β-Glucuronidase Inhibitors from the Stems of *Schisandra sphaerandra* Staph

**DOI:** 10.3390/ph15030329

**Published:** 2022-03-09

**Authors:** Guiwei Rao, Hangfei Yu, Manlai Zhang, Yuchen Cheng, Kun Ran, Jianwei Wang, Bin Wei, Min Li, Weiguang Shan, Zhajun Zhan, Youmin Ying

**Affiliations:** 1 College of Pharmaceutical Science, Zhejiang University of Technology, Hangzhou 310014, China; xiankelaifeng@163.com (G.R.); 2111907099@zjut.edu.cn (H.Y.); zhangmanlai@163.com (M.Z.); rk_124jy@163.com (K.R.); wangjianwei@zjut.edu.cn (J.W.); biwei@zjut.edu.cn (B.W.); tianranyaowu@zjut.edu.cn (W.S.); zgdnpr@zjut.edu.cn (Z.Z.); 2 Interdisciplinary Research Academy, Zhejiang Shuren University, Hangzhou 310015, China; 3 University of Edinburgh Institute, Zhejiang University, Haining 314400, China; yuchenc.18@intl.zju.edu.cn; 4 Zhejiang Huahai Pharmaceutical Co., Ltd., Taizhou 317000, China; mili1207@163.com

**Keywords:** α-glucosidase, bacterial β-glucuronidase, *Schisandra sphaerandra*, diabetes, drug-induced diarrhea, triterpenoids

## Abstract

α-Glucosidase (AGS) is a therapeutic target for Type 2 diabetes mellitus (T2DM) that tends to complicate with other diseases. Some medications for the treatment of T2DM complications have the risk of inducing severe adverse reactions such as diarrhea via the metabolism of intestinal bacterial β-glucuronidase (BGUS). The development of new AGS and/or BGUS inhibitors may improve the therapeutic effects of T2DM and its complications. The present work focused on the isolation and characterization of AGS and/or BGUS inhibitors from the medicinal plant *Schisandra sphaerandra*. A total of eight compounds were isolated and identified. Sphaerandralide A (**1**) was obtained as a previously undescribed triterpenoid, which may have chemotaxonomy significance in the authentication of the genus *Schisandra* and *Kadsura*. 2′-acetyl-4′,4-dimethoxybiphenyl-2-carbaldehyde (**8**) was obtained from a plant source for the first time, while compounds **2**–**7** were isolated from *S. sphaerandra* for the first time. In the in vitro assay, compounds **1**–**5** showed potent to moderate activity against AGS. Interestingly, compound **3** also exhibited significant BGUS inhibitory activity, demonstrating the potential of being developed as a bifunctional inhibitor that may find application in the therapy of T2DM and/or the diarrhea induced by medications for the treatment of T2DM complications.

## 1. Introduction

Diabetes mellitus (DM), a chronic metabolic disease characteristic of prolonged high blood sugar levels, has caused widespread concerns around the world. By 2040, the prevalence of DM is expected to reach 642 million [1]. Type 2 diabetes mellitus (T2DM), caused by impaired insulin secretion and insulin resistance, accounts for more than 90% of all the incidences and is the most prevalent form of DM [2]. α-Glucosidases (AGS) hydrolyze the α-glucopyranosidic bond in complex carbohydrates to release glucose and other monosaccharides, leading to elevated blood sugar levels [3]. The inhibition of AGS can delay the digestion of carbohydrates and diminish the absorption of monosaccharides, which renders it as an ideal target for the management of T2DM. In fact, the use of AGS inhibitors has been proven to be the most efficient remedy for the control of postprandial hyperglycemia in T2DM [3]. Currently, three AGS inhibitors, namely acarbose, miglitol and voglibose, are used in clinic. However, regular consumption of these drugs has been reported to cause various side effects [4]. Hence, researchers are still engaged in the discovery of novel bioactive AGS inhibitors.

DM patients usually suffer from complications such as foot ulcers, diabetic retinopathy, nephropathy, cardiovascular diseases, stroke, and neuropathy [5], which have been reported to be associated with the mild-to-moderate proinflammatory status in DM patients [6]. Preliminary results of clinical trials have opened the door for immunomodulatory strategies for the treatment of T2DM and the associated complications involving the use of non-steroidal anti-inflammatory drugs (NSAIDs) and interleukin-1 antagonists [7]. Moreover, consistent hyperglycemia and insulin resistance in T2DM have also been found to be related to the pathogenesis and development of colorectal cancer (CRC) [8,9,10,11]. A retrospective analysis revealed the prevalence of T2DM among CRC to be 8.04% [12]. Consequently, for T2DM patients complicated with cancer, chemotherapeutic drugs were usually prescribed in combination with hypoglycemic agents. Although Irinotecan (CPT-11) has been known as a first-line drug for the treatment of CRC, its efficacy is usually compromised by the severe side effect, diarrhea [13]. Recent studies have demonstrated the pivotal role of the intestinal bacterial β-glucuronidase (BGUS) in the onset of drug-induced diarrhea, since BGUS could significantly modulate the pharmacokinetic properties of drugs (such as CPT-11 and NSAIDs) by hydrolyzing the glucuronidated metabolites into the corresponding aglycones with strong toxicity [14,15]. The overproduction of the toxic aglycone in the intestine may then lead to severe gastrointestinal reactions. Therefore, BGUS has been regarded as a potential target for alleviating drug-induced gastrointestinal reactions, and the development of BGUS inhibitors are of vital importance for the amelioration of drug-induced life-threatening diarrhea.

The genus *Schisandra* comprises about 30 species. *S. chinensis* and *S. sphenanthera* are two representative species in the genus, fruits of which have been recorded in the Chinese Pharmacopoeia as two different original plants for the traditional Chinese medicine “wuweizi” [16]. Previous studies have revealed that plants of the genus *Schisandra* are rich sources of lignans [17] and triterpenoids [18,19]. *S.* *sphaerandra* Staph, distributed mainly in southern China, has been used as a folk medicine to treat stomach diseases [20]. As compared with those of *S. chinensis* and *S. sphenanthera*, phytochemical study of *S. sphaerandra* was limited. To date, only twenty-two triterpenoids, twelve lignans, one phenolic compound, two steroids, and two fatty acids have been obtained from this plant [20,21,22], since the discovery of nigranoic acid as an HIV-1 reverse transcriptase inhibitor from it in 1996 [20]. Among them, the preschisanartane-type schinortriterpenoids from *S. sphaerandra* were reported to possess neurite outgrowth-promoting and neural injury-protective activities [22].

As part of our ongoing efforts in exploring natural products for AGS [23,24,25,26] and/or BGUS inhibitors [27], the ethanol extract of *S. sphaerandra* (SSE) was found to inhibit the activities of both AGS and BGUS in vitro. Subsequent investigation led to the isolation and identification of twelve dibenzocyclooctene lignans characteristic of the genus *Schisandra* [28]. However, all the lignans were found to be inactive against either AGS or BGUS [28]. Discrepancy between the activities of SSE and the isolated lignans prompted us to proceed with further in-depth phytochemical studies on SSE. As a result, six triterpenoids **1**–**6**, one sesquiterpenoid **7**, and one biphenyl derivative **8**, were obtained from SSE; **1** was determined to possess a previously undescribed structure, while **8** was obtained from a plant source for the first time. In the in vitro assays, **1**–**5** exhibited potent inhibitory activity against AGS. Interestingly, **3** also significantly inhibited the activity of BGUS, showing the potential to be developed as a bifunctional inhibitor that may find application in the therapy of T2DM and/or the diarrhea induced by medications for the treatment of T2DM complications. This was the first time that *Schisandra* triterpenoids were reported as AGS and/or BGUS inhibitors. The present work reports the isolation, structure characterization, and biological evaluation of these compounds.

## 2. Results and Discussion

### 2.1. Structure Elucidation

**Figure 1:**
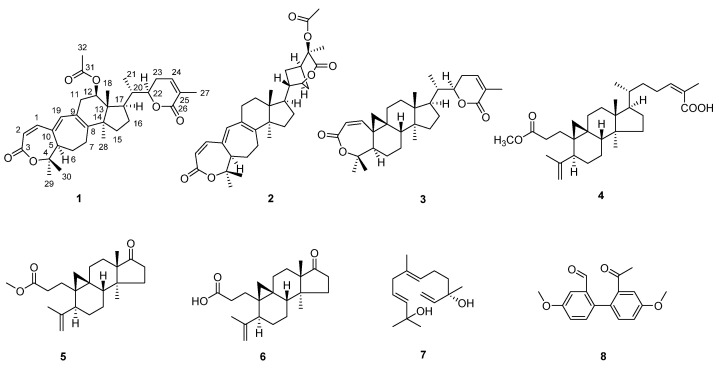
Chemical structures of **1−8**.

The 95% ethanol extract of the stems of *S. sphaerandra* was partitioned between water and EtOAc. Repeated column chromatography (CC) of the resulting EtOAc-soluble fraction afforded **1**–**8** (Figure 1).

**Figure 2:**
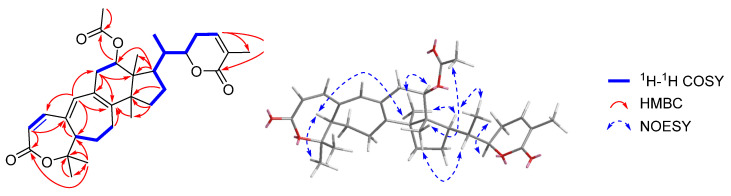
Key ^1^H-^1^H COSY, HMBC, and NOESY correlations with **1**.

Sphaerandralide A (**1**), colorless crystals with a mp of 242–243 °C, has a molecular formula of C_32_H_42_O_6_ as deduced from the HR-ESI-MS [M + H]^+^ iron at *m*/*z* 523.3049 (calculated for C_32_H_43_O_6_, 523.3054), corresponding to 12 degrees of unsaturation. The IR spectrum of **1** showed the presences of two lactones (1716 and 1686 cm^−1^). The ^1^H-NMR spectrum (Table 1, see Supplementary Materials) displayed signals for six methyl singlets at *δ*_H_ 0.87 (3H, s, CH_3_-18), 1.16 (3H, s, CH_3_-28), 1.39 (3H, s, CH_3_-30), 1.53 (3H, s, CH_3_-29), 1.91 (3H, s, CH_3_-27), and 2.06 (3H, s, CH_3_-32), one methyl doublet at *δ*_H_ 1.00 (3H, d, *J* = 6.8 Hz, CH_3_-21), two oxygenated methine protons at *δ*_H_ 4.51 (1H, dt, *J* = 12.9, 3.6 Hz, H-22) and 5.25 (1H, dd, *J* = 7.8, 7.8 Hz, H-12), and four olefinic protons at *δ*_H_ 5.83 (1H, d, *J* = 12.1 Hz, H-2), 6.17 (1H, s, H-19), 6.60 (1H, d, *J* = 6.3 Hz, H-24), and 6.65 (1H, d, *J* = 12.3 Hz, H-11). The ^13^C-NMR and DEPT spectra revealed the presence of thirty-two carbons comprised of seven methyls, six methylenes, nine methines (two oxygenated at *δ*_C_ 74.4 and 80.7, and four olefinic at *δ*_C_ 118.5, 139.4, 141.3, and 143.4), seven non-protonated carbons (one oxygenated at *δ*_C_ 80.4 and four olefinic at *δ*_C_ 128.4, 128.6, 140.3, and 149.5), and three carbonyls at *δ*_C_ 166.4, 167.0, and 170.9. The above-mentioned information suggested that **1** was a triterpenoid. After detailed analysis of the 2D-NMR spectra (Figure 2), **1** was identified to possess an A,B-seco-9,19-cyclolanostene skeleton with a seven-membered α,β-unsaturated lactone moiety in ring A and a six-membered α,β-unsaturated lactone moiety in the side chain, which was also supported by the UV spectrum [λ_max_ (logε): 326 (4.32), 211 (4.24)]. Additional HMBC correlation with H_3_-32 (*δ*_H_ 2.06)/C-31 (*δ*_C_ 170.9) evidenced the presence of an acetoxyl group, and it was deduced to anchor at C-12 by the HMBC correlation with H-12 (*δ*_H_ 5.25)/C-31 (*δ*_C_ 170.9). The planar structure of **1** was thus established, which was identical to that of heteroclitalactone D [29]. Nevertheless, obvious differences were observed for the chemical shifts of H-12 in the ^1^H NMR spectra of heteroclitalactone D (*δ*_H_ 4.97 in CDCl_3_) and **1** (*δ*_H_ 5.25 in CDCl_3_) after detailed comparison, proposing that **1** was the stereoisomer of heteroclitalactone D at C-12.

**Figure 3:**
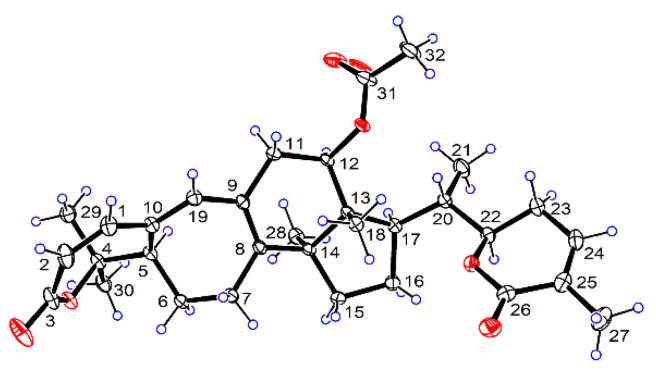
ORTEP diagram of **1**.

In the NOESY spectrum of **1** (Figure 2), correlations with H_3_-29/H-5, H-5/H_3_-28, H_3_-28/H-12, H-12/H-17, H-17/H_3_-21, and H_3_-21/H-22 suggested the *α*-orientation of these groups. Furthermore, key NOE correlations with H-20/H_3_-18 and H_3_-18/H_3_-32 suggested the *β*-orientation of the acetoxy group in **1**, in contrast to the α-oriented acetoxy group in heteroclitalactone D. Fortunately, we obtained the single crystal of **1** from a mixture of *n*-hexane/EtOAc/CH_2_Cl_2_ (2:1:1, *v*/*v*). Single-crystal X-ray diffraction experiment with Cu Kα radiation validated the planar structure and relative stereochemistry of **1** and designated its absolute configurations as *5R*,*12R*,*13R*,*14S*,*17R*,*20S*,*22R* (CCDC: 2130778) (Figure 3). Compound **1** was thus unambiguously identified to possess the structure as shown in Figure 1.

Seven known compounds were also isolated from the stems of *S. sphaerandra* in the present study. They were identified as henrischinin B (**2**) [30], schisanlactone B (**3**) [31], 3,4-seco-(24*Z*)-cycloart-4(28),24-diene-3,26-dioic-3-methyl ester (**4**) [32], schiglausin L (**5**) [33], micranoic acid B (**6**) [34], 3,6,11-dodecatriene-2,10-diol (**7**) [35], and 2′-acetyl-4′,4-dimethoxybiphenyl-2-carbaldehyde (**8**) (Figure 1) by comparing the NMR data with those reported in the literature [36].

The isolation and identification of **1**–**8** further expanded the chemical diversity of *S. sphaerandra*. According to the literature [29,37,38,39], heteroclitalactone D has been solely isolated from the genus *Kadsura* that was closely related to the genus *Schisandra*. Acquisition of the new triterpenoid sphaerandralide A from *S. sphaerandra* in the present study put forward the possibility that these two epimers may be of significance in the chemotaxonomy of *Schisandra* and *Kadsura*. Compounds **2**–**8** were all isolated from *S. sphaerandra* for the first time. In particular, it was the first time that compound **8** was obtained from a plant source.

### 2.2. AGS and BGUS Inhibitory Activity

Compounds **1**–**8** were evaluated for the in vitro AGS and BGUS inhibitory activities, respectively. In the AGS inhibitory assay, compounds **1**–**5** showed potent to moderate activity with IC_50_ values ranging from 14.08 ± 0.29 to 74.45 ± 1.13 µM (Table 2), as compared with acarbose (IC_50_ = 422.3 ± 8.44 μM). Compounds **1**–**3** possessed rearranged triterpenoid skeletons that were frequently encountered in plants of the genus *Schisandra* and *Kadsura*. Preliminary structure–activity relationship was also summarized. Specifically, compound **3** exhibited more potent activity than that of **1** and **2**, suggesting that the six-membered ring B and the fused cyclopropane ring may play a vital role in retaining the AGS inhibitory activity of this series of triterpenoids. Compound **4** was a 3,4-seco-cycloartane triterpenoid, while **5** and **6** belonged to 3,4-seco-cycloartane octanortriterpenoids. By comparing the activity of **4**–**6**, it was concluded that the existence of the side chain at C-17 contributed to the maintenance of the AGS inhibitory activity as in the case of **4** that exhibited potent activity. For the two 3,4-seco-cycloartane octanortriterpenoids, hydrolysis of the methyl ester at C-3 led to the loss of the activity.

In the BGUS inhibitory assay, **3** showed potent activity with an IC_50_ value of 14.70 ± 0.10 μM as compared with that of D-Saccharic 1,4-lactone (DSL) with an IC_50_ value of 56.85 ± 1.57 μM, while **4** showed weak activity (Table 2).

### 2.3. Analysis of Inhibition Kinetics

**Figure 4:**
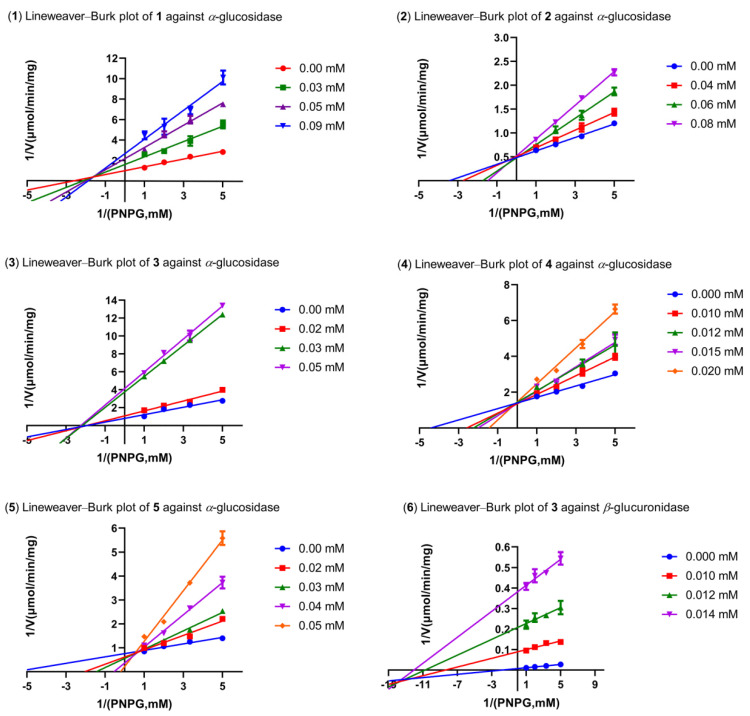
The Lineweaver–Burk plots of **1**–**5** against AGS and **3** against BGUS.

The inhibition kinetic mechanisms of **1**–**5** against AGS were studied using the Lineweaver–Burk plots (Figure 4). The inhibition constant of the enzyme K_i_ and the inhibition constant of the enzyme–substrate complex K_i’_ of the inhibitors (Table 3 and Table 4) were obtained by secondary plots of “slope *versus* [I]” and “Y-intercept *versus* [I]”, respectively. As shown in Figure 4, data lines of **1** intersected in the second quadrant, while those of **3** had intersections in the third quadrant. In addition, K_m_ and V_max_ values of both **1** and **3** changed with the increased concentration of inhibitors. These results suggested that both **1** and **3** were mixed-type inhibitors of AGS, indicating they were able to bind either the free AGS or the AGS-substrate complex. As for **1**, the inhibition constant K_i_ (35.78 μM) was smaller than K_i’_ (56.99 μM), demonstrating that it bound more easily and tightly to the free AGS than the AGS-substrate complex. On the contrary, **3** tended to bind more preferably to the AGS-substrate complex as indicated by a larger K_i_ (10.47 μM) than K_i′_ (7.93 μM). In addition, the smaller K_i_ and K_i’_ of **3** (10.47 and 7.93 μM, respectively) indicated better inhibitory potency against AGS as compared with that of **1** (35.78 and 56.99 μM, respectively), which was consistent with the IC_50_ values. Intersection of the data lines on the y-axis indicated that both **2** and **4** were competitive inhibitors of AGS, which was supported by the increased K_m_ and constant V_max_ values. By comparison with the Ki values of **4** (7.97 μM) and **2** (45.15 μM), **4** was proposed to be a more potent inhibitor of AGS, which was verified by the IC_50_ values in the AGS inhibition assay. The inhibition behavior of **5** could not be well defined since the Lineweaver–Burk plots intersected in the first quadrant. As a result, other kinetic parameters of **5** remained undetermined, except for the K_m_ values.

**Figure 5:**
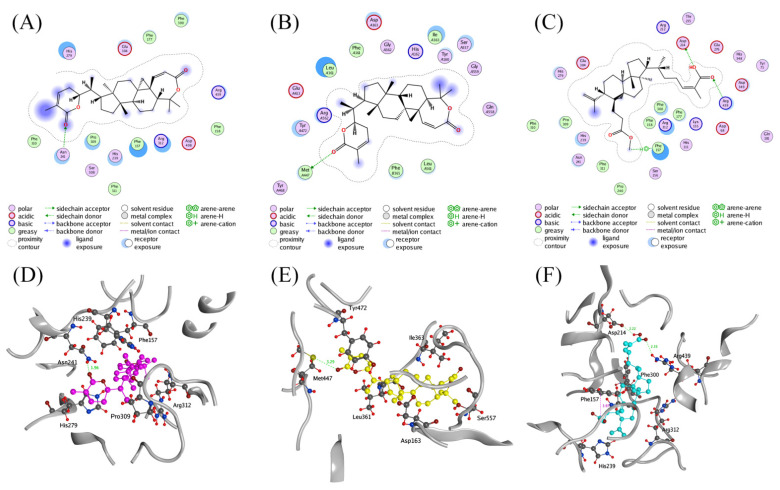
Ligand interactions of (**A**) 3 with AGS, (**B**) 3 with BGUS, and (**C**) 4 with AGS; binding modes of (**D**) 3 (magenta) with AGS, (**F**) 4 (blue) with AGS, and (**E**) 3 (yellow) with BGUS.

Compound **3** was found to be a mixed-type inhibitor of BGUS, since the straight lines on the Lineweaver–Burk plots intersected in the third quadrant (Figure 5). This inhibition mode against BGUS was also validated by the varied K_m_ and V_max_ values following concentration changes of **3** (Table 4). However, the replots of slope and Y-intercept versus the concentration of **3** were not linearly fitted, which limited the application of Equations (3) and (4). Consequently, the K_i_ and K_i′_ of **3** remained not calculated.

### 2.4. Molecular Docking Studies

Given the significant activity of **3** and **4**, docking studies were performed to illustrate the molecular determinants of these two compounds in inhibiting AGS or BGUs. The interactions between the ligands and AGS or BGUS were studied using MOE. As shown in Figure 5, both **3** and **4** could be well docked into the active site of AGS and/or BGUS; **3** formed a hydrogen bond interaction with Asn241 of AGS via the carbonyl of the six-membered lactone ring with a length of 1.96 Å. In addition, the complex of AGS and **3** was also stabilized by the hydrophobic interactions with residues such as His279, Pro309, Phe157, Arg312, and His239 (Figure 5A,D). Likewise, a significant hydrogen bond interaction between **3** and Met447 (3.29 Å) of BGUS could be observed in the complex of **3** and BGUS (Figure 5B,E), as well as hydrophobic interaction between **3** and residues Leu362, Ile363, Asp163, Ser557, and Tyr472 in BGUS. These interactions were proposed to contribute to the bifunctional inhibitory activity of AGS and BGUS by **3**.

In the case of **4**, it formed hydrogen bond interactions with Asp214 (2.22 Å) and Arg439 (2.33 Å) in AGS via the carboxyl group in the side chain. Furthermore, a hydrogen-π interaction was detected between Phe157 and the methoxy group of **4**. The presences of hydrophobic interactions with residues Arg312, Phe300, and His239 also stabilized the complex of AGS and **4** (Figure 5C,F).

In fact, *S. sphaerandra* has scarcely been subjected to modern pharmacological study. This was the first time that triterpenoids from *S. sphaerandra* were identified as AGS and/or BGUS inhibitors. Nevertheless, the in vivo efficacy, mode of action, toxicology, and pharmacokinetics of these bioactive triterpenoids remain to be explored, in spite of their encouraging in vitro enzyme inhibition activities. They are expected to improve postprandial hyperglycemia in vivo after an oral sucrose tolerance test, such as other natural terpenoids exhibiting in vitro AGS inhibitory activity and in vivo antidiabetic effects [40,41,42]. In addition to the potent AGS inhibition activity, **3** also significantly inhibited BGUS, demonstrating the potential to be developed as a bifunctional inhibitor. Since AGS and BGUS co-localized in the intestinal tract, it is reasonable to postulate that **3** may work as a bifunctional inhibitor by targeting both enzymes simultaneously in vivo. In view of the wide prevalence of T2DM, as well as the high incidence of T2DM complications and the relevant risk of drug-induced diarrhea, the discovery and development of AGS and BGUS bifunctional inhibitors may provide a novel therapeutic strategy for the disease. These findings may endow *S. sphaerandra*, a previously neglected plant, with important medicinal value.

## 3. Materials and Methods

### 3.1. General Experimental Procedure

A Rudolph Research Autopol III polarimeter was used for optical rotations (Rudolph Research Analytical, Hackettstown, NJ, USA). A Thermo Nicolet 6700 FT-IR microscope instrument was used for the IR spectra (Thermo Electron Corporation, Waltham, MA, USA). A TU-1900 ultraviolet spectrometer was used for the UV spectra (Beijing Persee General Instrument Co., Ltd., Beijing, China). For HR-ESI-MS, an Agilent-6210-LC/TOF mass spectrometer was used (Agilent Technologies, Inc., Santa Clara, CA, USA). For NMR, a Bruker Avance 600 spectrometer was used (Bruker Corporation, Billerica, MA, USA). An Agilent Xcalibur Atlas Gemini Ultra diffractometer was used for single-crystal X-ray diffraction (Agilent Technologies, Inc., Santa Clara, CA, USA). For melting points, the X-5 microscopic melting point apparatus was used (uncorrected) (Beijing Tech Instrument Co., Ltd., Beijing, China). MCI CHP 20P gel (75–150 μm, Tokyo, Japan), silica gel (300–400 mesh; Qingdao Marine Chemical Co., Ltd., Qingdao, China), ODS AQ C-18 gel (50 μm; YMC Co., Ltd., Kyoto, Japan), and Toyopearl HW-40F gel (Tosoh Corporation, Tokyo, Japan), were used for CC. Precoated GF254 silica gel plates (Qingdao Marine Chemical Co., Ltd., Qingdao, China) were used for thin layer chromatography. A SpectraMax Plus 384 microplate reader (Molecular Devices, San Jose, CA, USA) was used in the AGS and BGUS inhibition bioassays.

### 3.2. Plant Material

The plant was collected in 2018 at Liangwang mountain of Yunnan Province, China. It was identified as *S. sphaerandra* by Dr. Jun Zhang from Kunming Zhifen Biotech. Samples of the plant material (ID ZJUT-WWZ2018-01) were deposited at the College of Pharmaceutical Science, Zhejiang University of Technology, Hangzhou, China.

### 3.3. Extraction and Isolation

**Figure 6:**
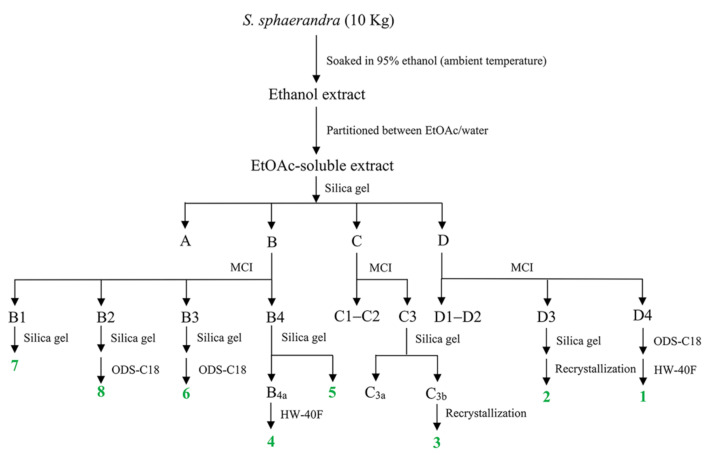
The separation scheme of **1**–**8**.

The stems of *S. sphaerandra* (dry weight 10 kg) were smashed and extracted with 95% ethanol (20 L, 3 days each) for three times under ambient temperature. The ethanol extract was concentrated and then partitioned between water and EtOAc (3 × 5 L). The EtOAc extract (108 g) was separated into four fractions (Fr. A–D) by CC on silica gel (petroleum ether (PE)-EtOAc (20:1 → 1:1, *v*/*v*)). Fr. B was fractionated by CC on MCI CHP 20P gel (CH_3_OH-H_2_O (65:35 → 90:10, *v*/*v*)) to yield four subfractions (Fr. B1–B4), and **7** (93.4 mg) was obtained from Fr. B1 (0.5 g) by CC on silica gel (PE-acetone (5:1, *v*/*v*)). Fr. B2 (156.7 mg) was subsequently purified on a silica gel column (PE-acetone (9:1, *v*/*v*)) followed by ODS AQ C-18 CC (CH_3_OH-H_2_O (80:20 → 100:0, *v*/*v*)) to give **8** (3 mg). Fr. B3 (776.5 mg) was successively chromatographed over silica gel CC (PE-acetone (10:1, *v*/*v*)) and ODS AQ C-18 CC (CH_3_OH-H_2_O (80:20 → 100:0, *v*/*v*)) to yield **6** (69.9 mg). Fr. B4 (1.47 g) was subjected to silica gel CC (PE-acetone (15:1, *v*/*v*)) to give **5** (7.4 mg) and another subfraction Fr. B4a. Fr. B4a (160.5 mg) was further purified by CC on Toyopearl HW-40F gel (CH_3_OH) to afford **4** (6.9 mg). Fr. C (20.2 g) was isolated by CC on MCI CHP 20P gel (CH_3_OH-H_2_O (70:30 → 90:10, *v*/*v*)) to yield three subfractions (Fr. C1–C3). Fr. C3 (1.89 g) was subjected to silica gel CC (PE-acetone (5:1, *v*/*v*)) to give two subfractions (Fr. C3a–C3b) and **3** (294.1 mg) was obtained from Fr. C3b (307.0 mg) by recrystallization in acetone. Fr. D (3.60 g) was separated by CC on MCI CHP 20P gel (CH_3_OH-H_2_O (80:20 → 90:10, *v*/*v*)) to offer four subfractions (Fr. D1–D4). Fraction D3 (2.04 g) was loaded onto silica gel CC (PE-acetone (4:1, *v*/*v*)), followed by recrystallization in CHCl_3_ to furnish **2** (1.2 g). Fraction D4 (1.20 g) was chromatographed by ODS AQ C-18 CC (CH_3_OH-H_2_O (80:20, *v*/*v*)) followed by purification on Toyopearl HW-40F gel (CH_3_OH) to obtain **1** (22.7 mg). The overall separation scheme is depicted in Figure 6.

Sphaerandralide A (**1**) were colorless crystals, mp 242–243 °C, ${\left[\alpha \right]}_{D}^{20}$: +168 (*c* 0.1, MeOH), IR (KBr): 2923, 1716, 1686, 1457, 1377, 1288, 1243, 1127, 1026, 986, 952, and 676 cm^−1^. UV λ_max_ (MeOH) nm (log*ε*): 326 (4.32) and 211 (4.24); HR-ESI-MS *m*/*z*: 523.3049 [M + H]^+^ (calculated for C_32_H_43_O_6_, 523.3054). For ^1^H- and ^13^C-NMR data, see Table 1.

### 3.4. Single-Crystal X-ray Diffraction

Single crystals of **1** were obtained from a mixture of *n*-hexane/EtOAc/CH_2_Cl_2_ (2:1:1, *v*/*v*) at 4 °C. The structures were solved with the ShelXT [43] program and refined with the ShelXL [44] refinement package using least squares minimization. Data were collected using Olex2 [45]. The crystallographic data for **1** (CCDC: 2130778) were deposited at the Cambridge Crystallographic Data Centre. The crystal structure of **1** was depicted in Figure 3.

*Crystallographic data for Sphaerandralide A* (**1**) were C_32_H_42_O_6_, M = 522.65 g/mol, orthorhombic, space group P212121 (no. 19), a = 7.3578(2) Å, b = 19.2407(5) Å, c = 19.7947(5) Å, *V* = 2802.32(13) Å^3^, *Z* = 4, *T* = 170.0 K, μ(CuKα) = 0.675 mm^−1^, *Dcalc* = 1.239 g/cm^3^, 27151 reflections measured (6.406° ≤ 2Θ ≤ 136.68°), 5143 unique (*R*_int_ = 0.0508, *R*_sigma_ = 0.0304), which were used in all calculations. The final *R*_1_ was 0.0349 (*I* > 2σ(I)) and *wR*_2_ was 0.0894 (all data). The goodness of fit on *F*^2^ was 1.052. The flack parameter = 0.08(5).

### 3.5. AGS and BGUS Inhibitory Assays

The AGS [23,24,25,26] and BGUS [27] inhibitory activities were evaluated by the methods reported in the literature, respectively.

### 3.6. Inhibition Kinetics Analysis

The mode of inhibition for the active compounds was determined by the Lineweaver–Burk plot fitted by GraphPad Prism 8.0 software. The K_m_ value and the V_max_ value were obtained from the slope and Y-axis intercept of the Lineweaver–Burk plot based on the following equation:

(1) $$V_{\max}=\frac{1}{Y-\mathrm{intercept}}$$

(2) $$K_{m}=\mathrm{slope}\times {\;V}_{\max}$$

The K_i_ and K_i’_ values were available by secondary plotting of the slope and Y-intercept on the Lineweaver–Burk plot versus the inhibitor [I] through the following equations:

(3) $$\mathrm{Slope}=\frac{K_{m}}{V_{\max}}+\frac{K_{m}\left[I\right]}{V_{\max}K_{i}}$$

(4) $$Y-\mathrm{intercept}=\frac{1}{V_{\max}}+\frac{\left[I\right]}{V_{\max}K_{i\prime }}$$

### 3.7. Molecular Docking Simulation

Inhibitors with significant potency were subjected to docking simulation, with the aim of revealing the probable molecular determinants underlying the inhibitory activity against AGS or BGUS. The X-ray crystal structure of BGUS (PDB ID: 3LPF) was obtained from the Protein Data Bank (PDB) database. Since the X-ray crystal structure of AGS of *Saccharomyces cerevisiae* has not been reported, a homology model of the enzyme was constructed by employing the crystal structure of isomaltase as a template (PDB ID: 3AJ7) on the SWISS MODEL webserver [46]. Then, the X-ray crystal structure of AGS built by homology modeling or BGUS was prepared using MOE (Version 2014. 09, Chemical Computing Group Inc., Montreal, Canada). The target compounds were docked into the active sites of AGS or BGUS using the Triangular Matching docking method. A total of 30 conformations for each ligand–protein complex were generated. Finally, the 2D and 3D plots were depicted for analysis of the interactions among inhibitors and the amino acid residues in the binding pocket.

## 4. Conclusions

Phytochemical studies on the stems of *S. sphaerandra* resulted in the isolation and identification of eight compounds. Sphaerandralide A (**1**) was obtained as a new triterpenoid, which may have chemotaxonomy significance in the authentication of the genus *Schisandra* and *Kadsura*. 2′-acetyl-4′,4-dimethoxybiphenyl-2-carbaldehyde (**8**) was obtained from a plant source for the first time. Compounds **2**–**7** were discovered from *S. sphaerandra* for the first time. In the in vitro AGS inhibition assay, compounds **1**–**5** showed potent to moderate activity. Inhibition kinetic studies revealed that **1** and **3** were mixed-type inhibitors, while **2** and **4** were competitive inhibitors against AGS. In particular, **3** also significantly inhibited the activity of BGUS in vitro in a mixed-type inhibition mode, demonstrating the potential to be developed as a bifunctional inhibitor that may find application in the therapy of T2DM and/or the diarrhea induced by medications for the treatment of T2DM complications.

## Figures and Tables

**Table 1:** ^1^H- (600 MHz) and ^13^C-NMR (150 MHz) spectroscopic data for **1** in CDCl_3_.

Position	*δ*_C_, Type	*δ*_H_ (Multi, *J* in Hz)
1	143.4	6.65 (d, 12.3)
2	118.5	5.83 (d, 12.1)
3	167.0	-
4	80.4	-
5	49.3	2.46 (dd, 12.5, 5.9)
6	39.1	2.37 (m) 2.24 (m)
7	27.6	1.92 (m) 2.09 (m)
8	149.5	-
9	128.6 ^Δ^	-
10	140.3	-
11	35.1	2.79 (ddd, 18.1, 7.7, 2.3) 2.00 (m)
12	74.4	5.25 (dd, 7.8, 7.8)
13	48.1	-
14	55.0	-
15	31.2	1.43 (m) 1.79 (m)
16	26.8	1.77 (m) 1.63 (m)
17	47.5	1.84 (m)
18	11.1	0.87 (s)
19	141.3	6.17 (s)
20	38.4	2.07 (m)
21	15.5	1.00 (d, 6.8)
22	80.7	4.51 (dt, 12.9, 3.6)
23	24.4	2.37 (m) 2.11 (m)
24	139.4	6.60 (m)
25	128.4 ^Δ^	-
26	166.4	-
27	17.0	1.91 (s)
28	26.9	1.16 (s)
29	26.3	1.53 (s)
30	29.3	1.39 (s)
31	170.9	-
32	21.8	2.06 (s)

^Δ^ Overlapped signals are reported without designated multiplicity.

**Table 2:** AGS and BGUS inhibitory activities of **1**–**8**.

Compounds	IC_50_ (μM) ^a^	
	α-Glucosidase	β-Glucuronidase
**1**	61.46 ± 1.75	NA ^a^
**2**	74.45 ± 1.13	NA
**3**	29.49 ± 0.70	14.70 ± 0.10
**4**	14.08 ± 0.29	154.6 ± 4.68
**5**	42.94 ± 1.04	NA
**6**	NA ^b^	NA
**7**	NA	NA
**8**	NA	NA
acarbose	422.3 ± 8.44	-
DSL	-	56.85 ± 1.57

^a^ Data are presented as means ± SD; ^b^ NA: no activity.

**Table 3:** Inhibition kinetics of **1**–**5** against AGS.

Compounds	Concentration (mM)	K_m_ (mM)	K_i_ (μM)	K_i′_ (μM)
**1**	0.00	0.38 ± 0.06	35.78	56.99
	0.03	0.47 ± 0.06		
	0.05	0.50 ± 0.09		
	0.09	0.53 ± 0.09		
**2**	0.00	0.29 ± 0.02	45.15	-
	0.04	0.36 ± 0.04		
	0.06	0.58 ± 0.06		
	0.08	0.68 ± 0.02		
**3**	0.00	0.51 ± 0.06	10.47	7.93
	0.02	0.51 ± 0.06		
	0.03	0.46 ± 0.01		
	0.05	0.44 ± 0.04		
**4**	0.00	0.23 ± 0.02	7.97	-
	0.01	0.38 ± 0.04		
	0.012	0.44 ± 0.07		
	0.015	0.50 ± 0.07		
	0.02	0.69 ± 0.08		
**5**	0.00	0.18 ± 0.02	NC ^a^	-
	0.02	0.49 ± 0.04		
	0.03	0.68 ± 0.05		
	0.04	1.81 ± 0.19		
	0.05	4.73 ± 0.31		

^a^ NC: not calculated.

**Table 4:** Inhibition kinetics of **3** against BGUS.

Compound	Concentration (mM)	K_m_ (mM)	K_i_ (μM)	K_i′_ (μM)
**3**	0.00	0.55 ± 0.04	NC ^a^	NC
	0.01	0.12 ± 0.03		
	0.012	0.09 ± 0.03		
	0.014	0.08 ± 0.02		

^a^ NC: not calculated.

## Data Availability

Data is contained within the article and Supplementary Materials.

## Supplementary Materials

The following supporting information can be downloaded at: https://www.mdpi.com/article/10.3390/ph15030329/s1, Figure S1: ^1^H-NMR spectrum of **1**, Figure S2: ^13^C-NMR spectrum of **1**, Figure S3: HSQC spectrum of **1**, Figure S4: HMBC spectrum of **1**, Figure S5: DEPT spectrum of **1**, Figure S6: ^1^H-^1^H COSY spectrum of **1**, Figure S7: NOESY spectrum of **1**, Figure S8: The IR (KBr disc) spectrum of **1**, Figure S9: HR-ESI-MS spectroscopic data for **1**, and Figure S10: UV spectrum of **1**.
