# Supplementary material for: α-Glucosidase and Bacterial β-Glucuronidase Inhibitors from the Stems of Schisandra sphaerandra Staph
Source: Pharmaceuticals (Basel). 2022 Mar 9;15(3):329. doi: 10.3390/ph15030329 (PMC8954508; doi:10.3390/ph15030329)
Supplement: Supplementary file 1 [file pharmaceuticals-15-00329-s001.zip › pharmaceuticals-1609676-supplementary.pdf]

pharmaceuticals

Supplementary Material

Alpha-glucosidase and Bacterial Beta-glucuronidase Inhibitors from the Stems of *Schisandra sphaerandra*

Guiwei Rao [1,2,#], Hangfei Yu [1,#], Manlai Zhang; [1] Yuchen Cheng; [3] Kun Ran [1], Jianwei Wang [1], Bin Wei [1], Min Li [4], Weiguang Shan [1], Zhajun Zhan [1], Youmin Ying [1,*]

1 College of Pharmaceutical Science, Zhejiang University of Technology, Hangzhou 310014, China;
2 Interdisciplinary Research Academy, Zhejiang Shuren University, Hangzhou 310015, China;
3 University of Edinburgh Institute, Zhejiang University, Haining, 314400, China;
4 Zhejiang Huahai Pharmaceutical Co. Ltd., Taizhou, 317000, China;
* Correspondence: ymying@zjut.edu.cn;
\# These authors contributed equally;

CONTENTS

Figure S1. ^{1}H NMR spectrum of **1** in $CDCl_3$ (600 MHz) 2
Figure S2. ^{13}C NMR spectrum of **1** in $CDCl_3$ (125 MHz) 2
Figure S3. HSQC spectrum of **1** in $CDCl_3$ 3
Figure S4. HMBC spectrum of **1** in $CDCl_3$ 3
Figure S5. DEPT spectrum of **1** in $CDCl_3$ 4
Figure S6. ^{1}H-^{1}H COSY spectrum of **1** in $CDCl_3$ 4
Figure S7. NOESY spectrum of **1** in $CDCl_3$ 5
Figure S8. The IR (KBr disc) spectrum of **1** 5
Figure S9. The (+)-HRESIMS spectroscopic data of **1**........ 6
Figure S10. UV spectrum of **1** 6

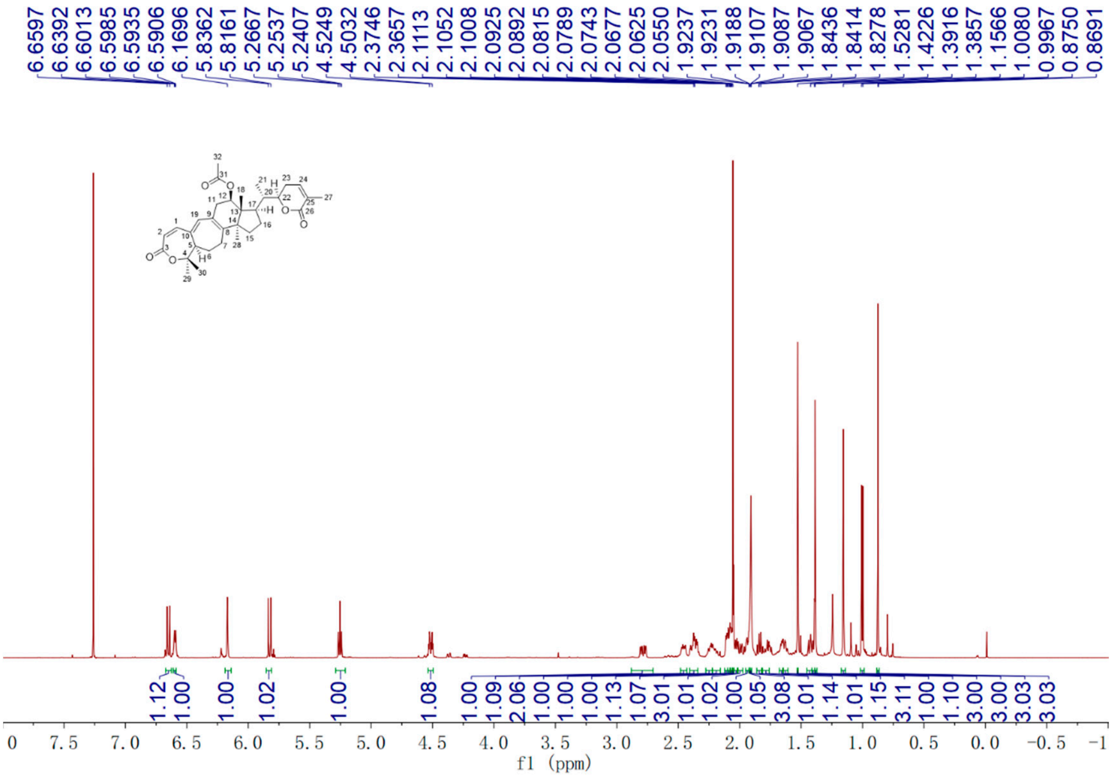

Figure S1. ^{1}H NMR spectrum of **1** in $CDCl_3$ (600 MHz)

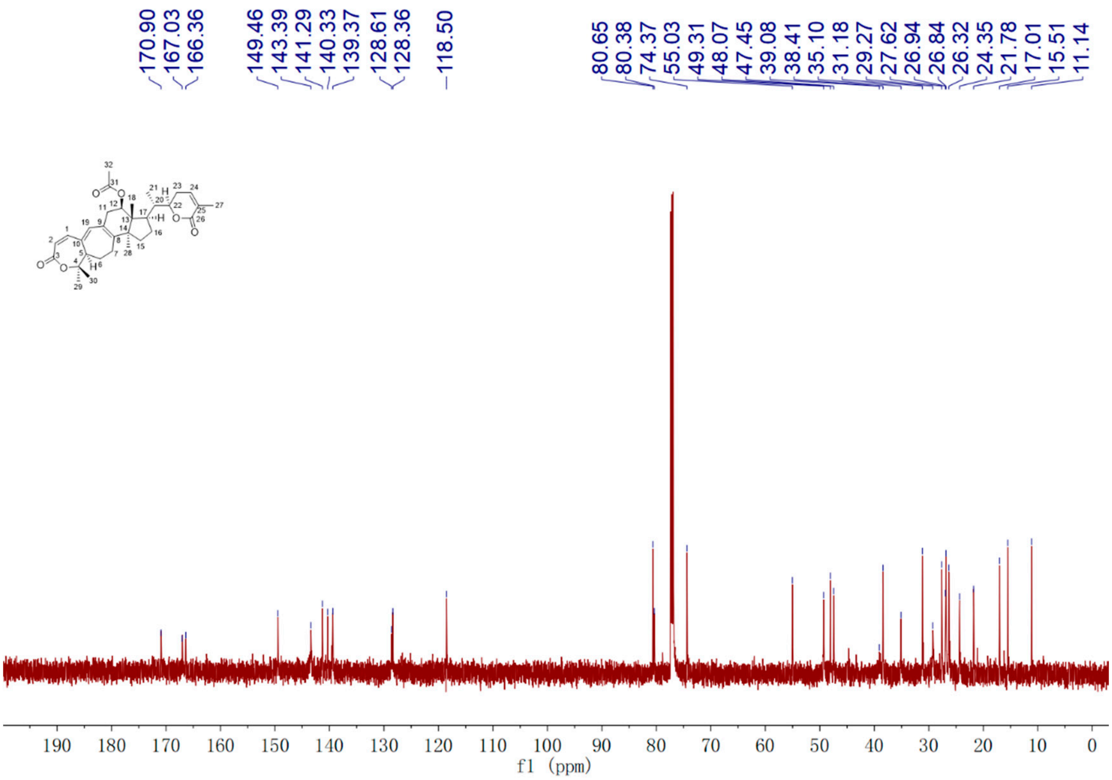

Figure S2. ^{13}C NMR spectrum of **1** in $CDCl_3$ (125 MHz)

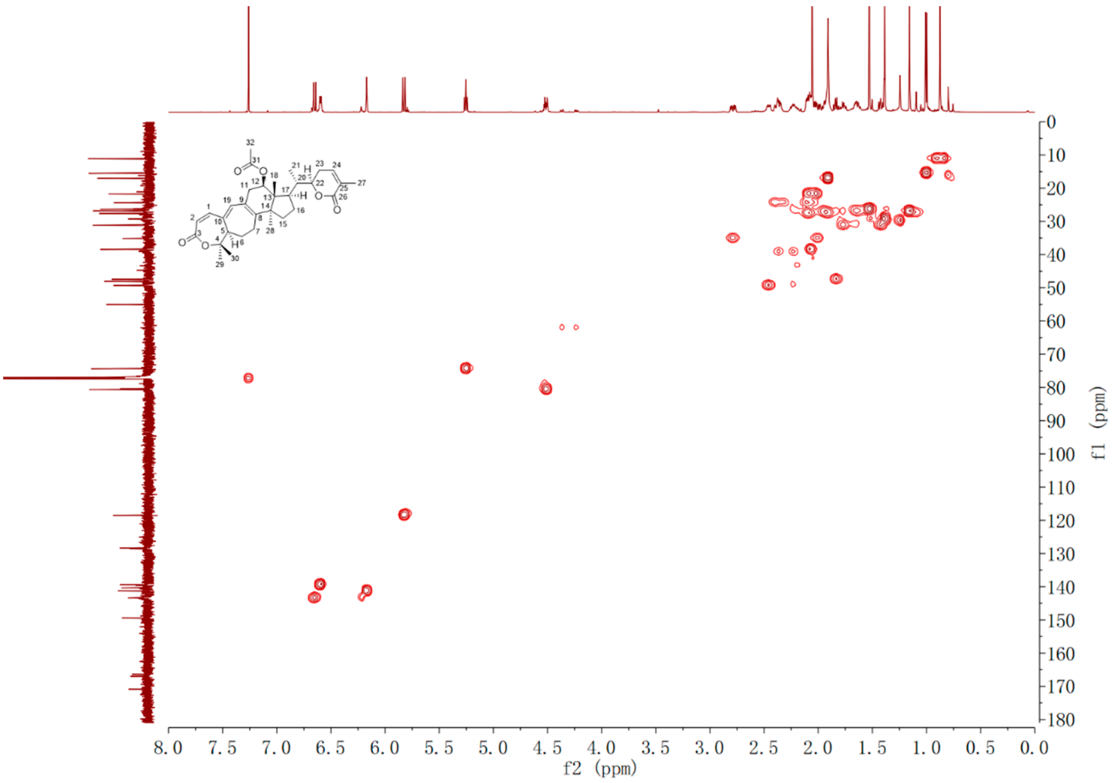

Figure S3. HSQC spectrum of **1** in $CDCl_3$

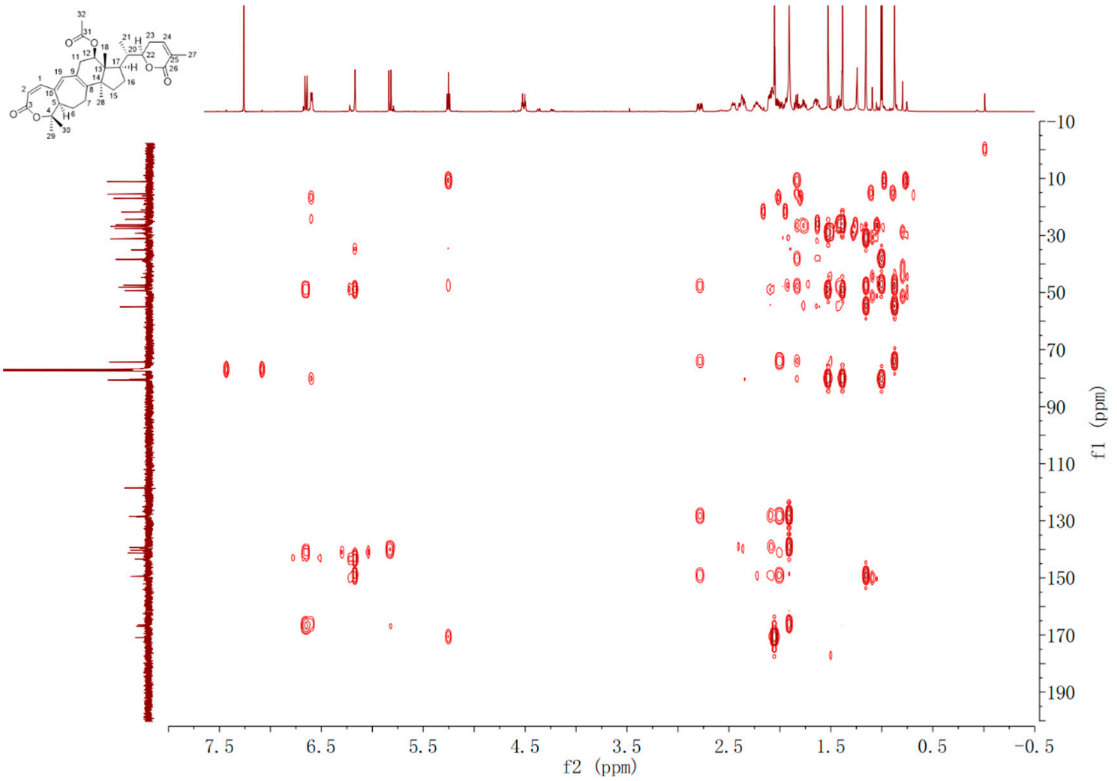

Figure S4. HMBC spectrum of **1** in $CDCl_3$

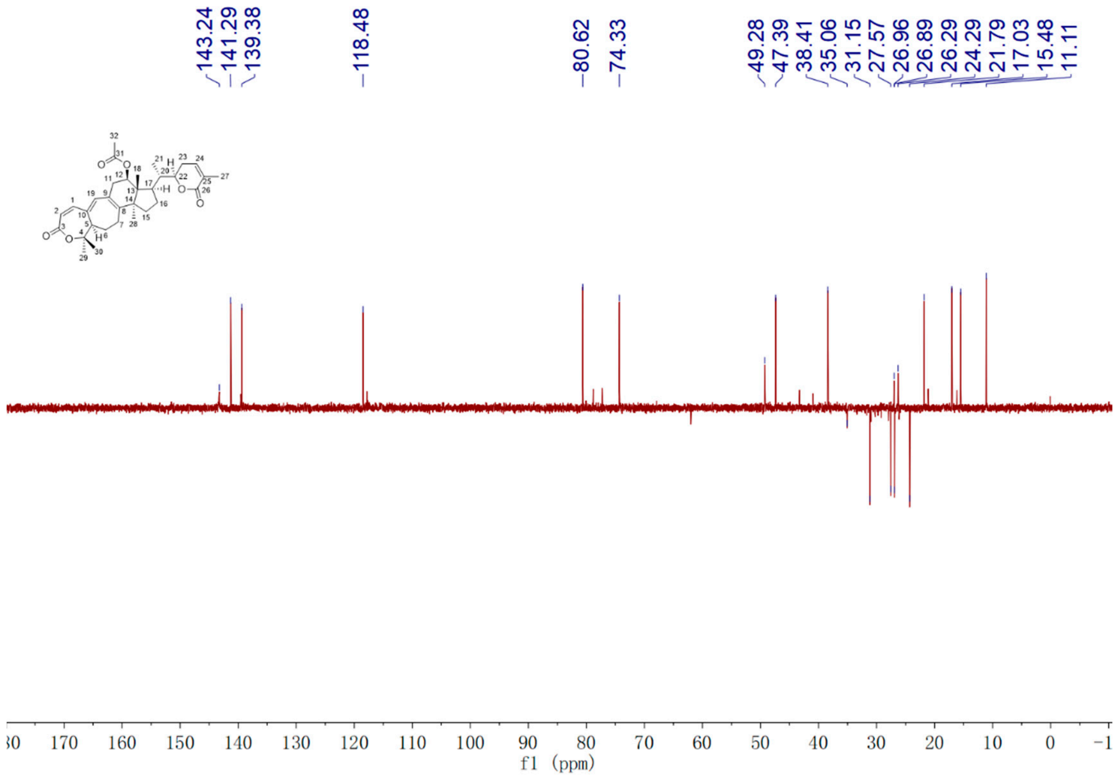

Figure S5. DEPT spectrum of **1** in CDCl3

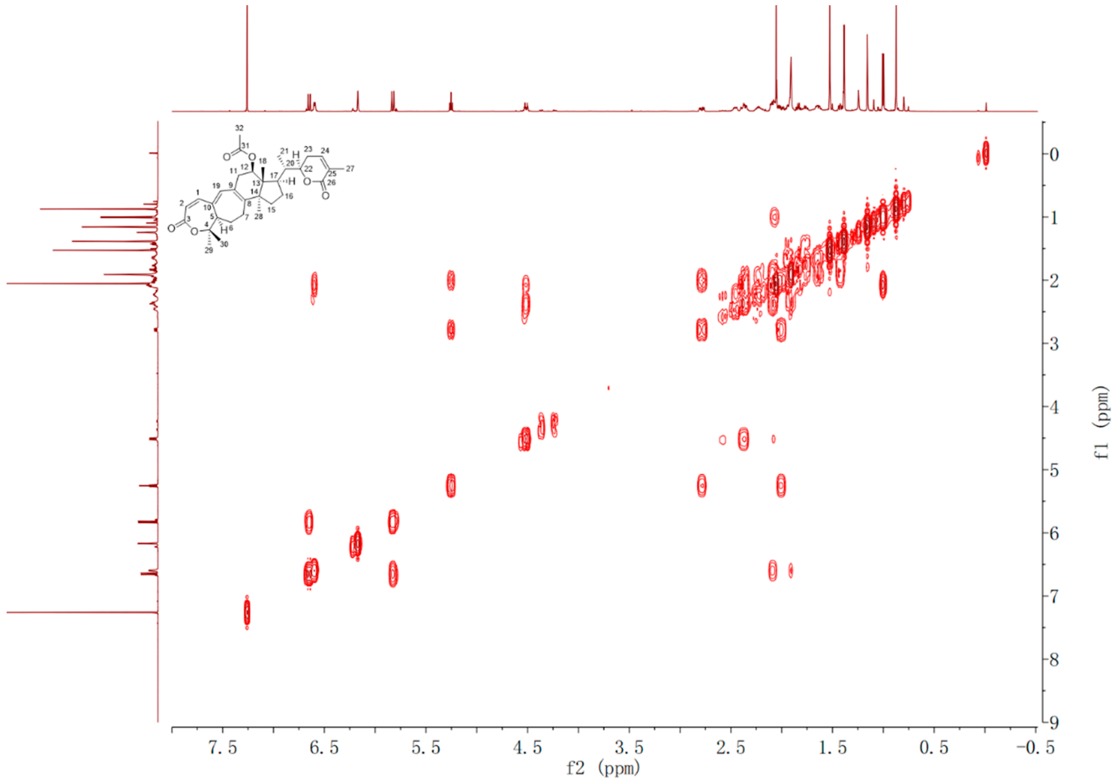

Figure S6. ^{1}H-^{1}H COSY spectrum of **1** in CDCl3

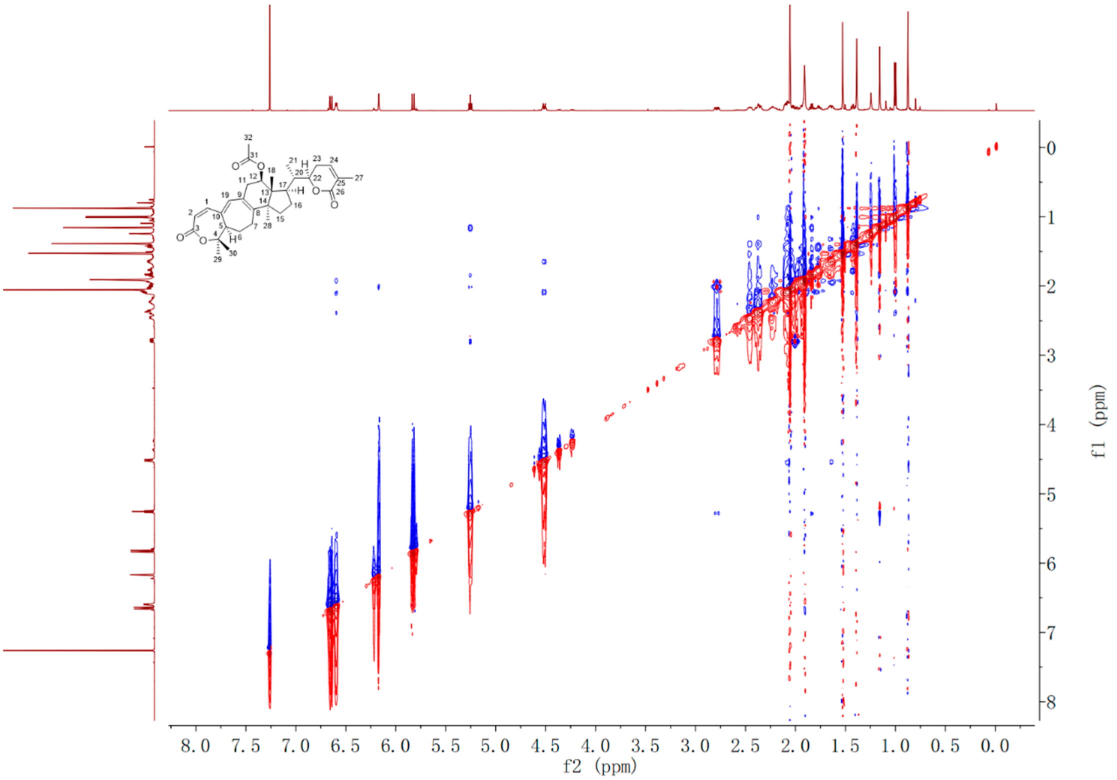

Figure S7. NOESY spectrum of **1** in $CDCl_3$

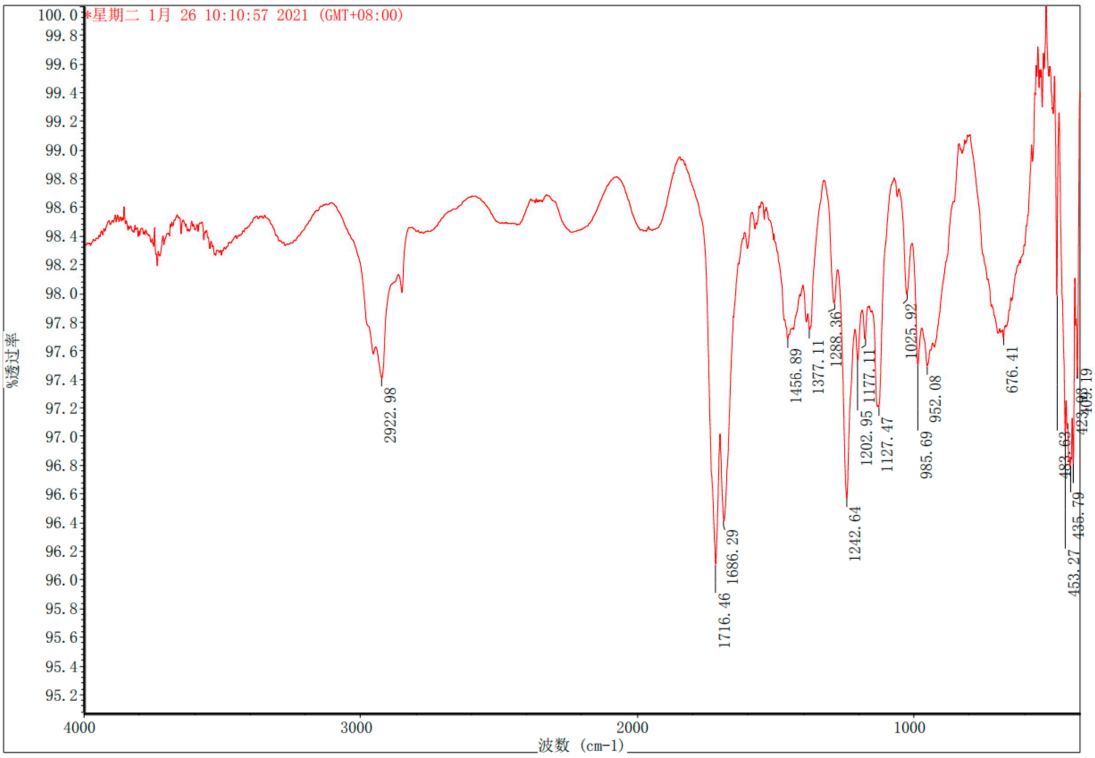

Figure S8. The IR (KBr disc) spectrum of **1**

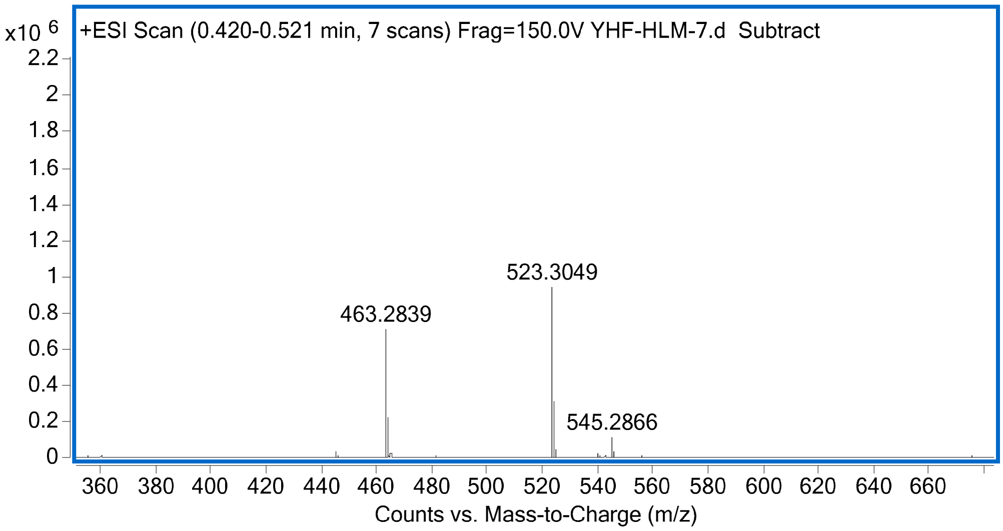

Formula (M)	Ion Formula	m/z	Calc m/z	Diff (ppm)	DBE
$C_{32}H_{42}O_6$	$C_{32}H_{43}O_6$	523.3049	523.3054	0.99	12

Figure S9. The (+)-HRESIMS spectroscopic data of **1**

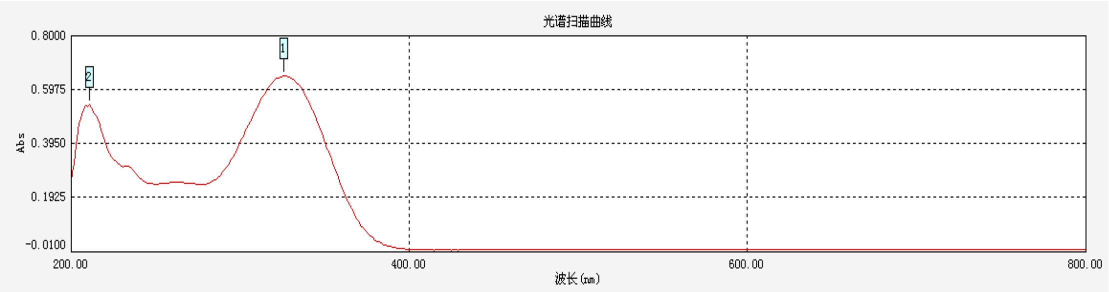

P	λ (nm)	Abs
1	326	0.6510
2	211	0.5420

Figure S10. UV spectrum of **1**
